# Blood pressure and cholesterol level checks as dynamic interrelated screening examinations

**DOI:** 10.1038/s41598-017-12904-4

**Published:** 2017-10-16

**Authors:** Alexander Labeit, Abbi Kedir, Frank Peinemann

**Affiliations:** 1 School of Health and Related Sciences, University of Sheffield, Sheffield, UK; 2 Centre for Applied Health Economics, School of Medicine, Griffith University, Nathan Campus, Australia; 3 Menzies Health Institute Queensland, Griffith University, Nathan Campus, Australia; 4 Management School, University of Sheffield, Sheffield, UK; 5 FOM University of Applied Science for Economics & Management, Essen, Germany; 6 Children’s Hospital, University Hospital of Cologne, Cologne, Germany

## Abstract

This study analysed the determinants of screening uptake for blood pressure and cholesterol level checks. Furthermore, it investigated the presence of possible spillover effects from one type of cardiovascular screening to another type of cardiovascular screening. A dynamic random effects bivariate panel probit model with initial conditions (Wooldridge-type estimator) was adopted for the estimation. The outcome variables were the participation in blood pressure and cholesterol level checks by individuals in a given year. The balanced panel sample of 21,138 observations was constructed from 1,626 individuals from the British Household Panel Survey (BHPS) between 1996 and 2008. The analysis showed the significance of past screening behaviour for both cardiovascular screening examinations. For both cardiovascular screening examinations state dependence exist. The study also shows a significant spillover effect of the cholesterol level check on the blood pressure check and vice versa. Also a poorer health status led to a higher uptake for both types of screening examinations. Changes in recommendations have to consider the fact that taking part in one type of cardiovascular screening examination can influence the decision to take part in the other type of cardiovascular screening examination.

## Introduction

Individuals can take part in different health screening examinations in the UK. These screening examinations include blood pressure check, cholesterol level check, dental screening, eyesight test and for women also breast cancer screening and cervical cancer screening. Blood pressure check and cholesterol level check are two cardiovascular screening examinations which are recommended according to the relevant medical guidelines^1,2^ and are often done, because of the high prevalence of cardiovascular diseases in the UK^3,4^. There is some empirical evidence that a positive association between different screening examinations exist^5,6^. However, there is no empirical analysis with longitudinal data which has investigated the uptake of two different cardiovascular screening examinations such as the blood pressure check and cholesterol level check over a long time period. Possible spillover effects could exist from one type of cardiovascular screening examination to the other type of cardiovascular screening examination and so they could influence each other in the uptake behaviour. First, the recommendations for the blood pressure check and cholesterol level check are described. Second, economic models and the relevance for prevention activities and potential weaknesses of the economic models are discussed. Third, hypotheses and empirical evidence for analysed variables are discussed. Fourth, the results are presented and discussed with potential implications for improving individuals’ uptake of the two screening examinations at the same time.

The National Health Service (NHS) and other medical organisations in the UK have published recommendations for blood pressure checks and cholesterol level checks in the relevant analysed time period^7–10^. The blood pressure can be checked by a GP or another healthcare professional, and it is recommended that adults aged over 20 are checked at least every 5 years and the recommendations for regular blood pressure are dependent on age, health status and existing diseases, health behaviour and lifestyle^7^. For older individuals and individuals with risk factors such as overweight, obesity, diabetes, family history of high blood pressure and smoking blood pressure should be checked every year and for individuals with existing hypertension blood pressure should be checked several times a year^8^. A cholesterol level check is recommended for adults with no symptoms to take place every 5 years starting at age 20^9,10^. It is recommended to do this test every 1–2 years for all individuals who are overweight or obese and have high blood pressure or diabetes or who have been diagnosed with coronary heart disease, stroke, peripheral arterial disease or who have a family history of early cardiovascular disease or a close family member with a cholesterol-related condition, such as familial hypercholesterolemia. The cholesterol level check is implemented as an invitational programme.

The recommendations of both health check-ups are relevant for analysing the uptake of both screening examinations, because there is an increased likelihood of participating in screening examinations after the recommended time interval. For both screening examinations, a borderline level of the blood pressure or cholesterol level in the actual year can induce a follow-up control screening examination in the next year.

The purpose of this study is to analyse how the blood pressure check and cholesterol level check influence as screening examinations each other and if spillover effects exist. A conceptual theoretical framework for the empirical analysis is used which includes economic and non-economic factors.

## Results

### Economic models and existing empirical evidence

Economic models for the demand of health care in general and for preventive services in particular are based on human capital models^11^. This theoretic framework has been used for modelling the demand of primary and secondary prevention^12^, because health check-ups have a self-protection function and improve early detection of diseases^13^. Both cardiovascular screening examinations are good examples of fulfilling a self-protection function and also of improving the early detection of the diseases such as hypertension and hypercholesterolemia. Relevant economic models for this analysis should make the distinction between acute and preventive care and should consider uncertainty in outcomes at the same time. Acute care describes the consumption aspect of health, whereas preventive care describes the investment aspect. Some dynamic economic models for the demand of health care take only uncertainty into consideration. However, there is no distinction made between acute and preventive care^14^. Other economic models such as the Grossman model makes the distinction between acute and preventive care, but these models do not consider uncertainty^15^. There is only one economic model which incorporates acute and preventive care and uncertainty in one model and which explicitly models the demand for preventive health care and uncertainty in a stochastic dynamic framework^16^. All the aforementioned economic models have the disadvantage of not considering non-economic factors. It is also of relevance to analyse non-economic factors for the uptake of screening examinations, because two reviews have shown their relevance for the uptake of screening examinations^17,18^. Many studies do not analyse non-economic factors in detail. The conceptual framework of the analysis is based on a human capital approach and the inclusion of non-economic factors.

### Hypotheses

There are different influence channels for the effect of age, education and household income on the demand for preventative services. It is known that age can have different effects on the demand for blood pressure check and cholesterol level check^19,20^. With respect to blood pressure check and cholesterol level check, recommendations exist for the screening examinations. On the one hand, according to the Grossman model health depreciates as the age increases and also the prevalence of hypertension and hypercholesterolemia increases with higher age^21,22^. Therefore, there is a higher need to maintain the health stock and as a consequence, the demand for prevention activities, such as blood pressure check and cholesterol level check increases with age. On the other hand, older individuals have a shorter life span and pay-off period for their investment in blood pressure check and cholesterol level check. Therefore, the effect of increasing age on the uptake for both screening examinations cannot be predicted. Empirical studies often find positive relationship between age and uptake for blood pressure check and cholesterol level check^23^. A higher educational level may be expected to lead to an increase in the uptake for blood pressure check and cholesterol level check, because individuals with a higher education level have a higher efficiency of health production, better capability of processing beneficial health advice, self-efficacy, motivation, awareness and knowledge about the importance of prevention including cardiovascular screening examination^12,24^. A higher household income leads to an increase in demand of time in perfect health and therefore the demand for both cardiovascular screening examinations should increase^11^. However, it was found that the effect of increasing household income on blood pressure check and cholesterol level check uptake was heterogeneous in two studies^20,25^. It can be expected that the effect of increasing household income on cardiovascular screening examinations should be either weaker or not existent in Great Britain when compared to other countries, because both cardiovascular screening examinations are free of charge in Great Britain.

The chance that an individual will visit a blood pressure check and cholesterol level check examination is dependent on a number of non-economic factors such as previous screening history, individual and household characteristics. We briefly discuss the existing empirical evidence in this paragraph. The history of previous screening examinations has a positive predictive value for uptake in the recent period, i.e. the past screening behaviour is correlated with the current behaviour and this result has been demonstrated both for non-cardiovascular screening examinations and cardiovascular screening examinations^17,23,24,26^. Cohabitation status is an indicator of a social support network and individuals living in a partnership are potentially better able to exchange information with their partners about health check-ups^27,28^. One study included the number of children as a variable and found that individuals with a higher number of children attended cervical cancer screening examinations less often^29^; however another study found no correlation with the number of children in a given household^30^. Employment was also added as a variable, because persons who work could have higher opportunity costs of time in comparison to unemployed and retired persons. A systematic review which analysed the influence of different determinants on the uptake of different health check-ups found that the influence of employment on the uptake of blood pressure check and cholesterol level check was varying in the analysed studies^17^. Several empirical analyses have shown that women have a lower uptake for cholesterol checks than men^31–33^. The GP plays a role as gatekeeper in the health care system and can give advice and information about the importance of blood pressure check and cholesterol level check examinations. Thus, the uptake of both cardiovascular screening examinations should be enhanced by previous GP visits^34,35^. Registration with a GP is a necessary condition for receiving an invitation letter for the cholesterol level check. Change of residence with the consequence of a new address lowers the chance of an individual to receive an invitation letter for the cholesterol level check. Poor self-perceived general health status should increase participation in cardiovascular screening examinations, because it offers a possibility to investigate the reason for the poor health status^20^. However, individuals with poor health status may be unable to visit the screening location such as the GP, because of acute and chronic diseases and other physical limitations. Hence, there are mixed results for the effect of poor health status or comorbidities on the uptake behaviour: in one study a higher comorbidity index led to lower screening rates of blood pressure check and the cholesterol level check^36^. However, it was found in two other studies that mammography utilization was higher among women with 3 or more stable comorbidities than among those without comorbidities^37,38^. Hypertension and diabetes mellitus should lead to an increase in both cardiovascular screening examinations, because they are recommended for individuals with both comorbidities. There is some evidence that elevated blood pressure is associated with elevated atherogenic blood lipid fractions such as the cholesterol level, but the association is especially weak for lean individuals and analyses of epidemiological surveys give inconsistent results across different population subgroups^39–42^.

Smoking can serve as an indicator for the weakened preference for health in comparison to other goods and individuals who smoke show a higher health risk taking behaviour^43,44^. Individuals who smoke have also poorer preventive health habits such as a reduced level of physical activity in comparison to non-smoking individuals^45^ and a reduced health care utilisation^46^. Ethnicity was added as a control variable, because ethnicity can have an influence on the probability of uptake of cardiovascular screening examinations and type of monitoring^47,48^.

### Analytical results

The balanced panel for blood pressure check and cholesterol level check consisted of 1,626 individuals with 21,138 observations from 1996 to 2008. Table 1 shows the proportion of individuals who had both cardiovascular screening examinations between 1996 and 2008 for every year. The uptake rate was 52.61% for blood pressure check and 21.42% for cholesterol level check over the whole period. Table 2 presents descriptive statistics for the balanced panels of both screening examinations.

**Table 1:** Uptake rate for blood pressure check and cholesterol level check during the 13 years period from 1996 to 2008.

**Health check-up**	**Blood pressure check**	**Cholesterol level check**
1996	44.83%	9.23%
1997	44.53%	11.32%
1998	46.56%	10.46%
1999	47.85%	12.30%
2000	45.57%	14.08%
2001	52.21%	16.85%
2002	53.20%	20.73%
2003	53.63%	24.05%
2004	57.6%3	28.04%
2005	57.81%	30.32%
2006	59.59%	32.10%
2007	60.02%	33.70%
2008	60.52%	35.30%
**Total**	52.61%	21.42%

Source: BHPS. The balanced panel consisted of 1,626 individuals with 21,138 observations.

**Table 2:** Descriptive characteristics for the balanced panel of blood pressure check and cholesterol level check.

Health check-up	Frequency or mean/SD
Total equivalised and deflated HH annual income (mean/SD)	3.01/(1.88)
Living with partner	0.746
Number of children in household (mean/SD)	0.506
Secondary education (ISCED)	0.412
Tertiary education (ISCED)	0.318
Employed part-time or full-time	0.531
GP visit during last 12 months	0.764
Health status good	0.468
Health status fair	0.247
Health status poor	0.077
Health status very poor	0.019
Status smoking	0.176
Moved residence within Great Britain	0.050
Region Scotland	0.078
Region Wales	0.050
Ethnic non-white	0.012
Age (mean/SD)	54.07/(15.96)
Female sex	0.586
Blood pressure problems	0.258
Diabetes mellitus	0.057

Source: BHPS. The balanced panel consisted of 1,626 individuals with 21,138 observations.

The results for the univariate pooled probit model and the dynamic random effects (RE) panel probit model with initial conditions (Wooldridge estimator) are given in Tables 3 and 4. All time varying variables were averaged over the panel period and used in the auxiliary regressions for the Wooldridge-type estimators for determining their effect on the individual specific term. The estimation used the same balanced sample of 1,626 individuals with 21,138 observations. For both cardiovascular screening examinations, taking part in a screening examination one, two and three years before showed a strong positive influence on the current screening examination suggesting a presence of state dependence. These results were similar in the univariate pooled and dynamic RE panel probit for the blood pressure check and cholesterol level check. For the univariate Wooldridge-type estimator, the coefficient was 0.480 for the first order own-effect lagged dependent variable, 0.217 for the second order own-effect lagged dependent variable and 0.186 for the third order own-effect lagged dependent variable in the blood pressure check equation. Similar results with a positive significant influence were found for the first, second and third order own-effect lagged dependent variable coefficients with values 0.926, 0.356 and 0.230 for the cholesterol level check equation. Additionally, there were cross-lagged dependent variable effects (spillover effects) for both types of screening examinations. It can be seen from Tables 3 and 4 that the coefficients for the univariate pooled probit and the dynamic random effects univariate panel probit model with initial conditions for blood pressure check and cholesterol level check were of similar size and the coefficients for the own-lagged dependent variables in the univariate pooled probit model were higher and overstating the state dependence. A comparison of the results for the unbalanced and balanced panels for the pooled univariate probit and the univariate Wooldridge-type estimators showed similar results for both types of cardiovascular screening examinations (results for the unbalanced panel estimations are available on request). This shows the robustness of our findings.

**Table 3:** Estimates of the univariate pooled and dynamic RE panel probit blood pressure check model.

	Univariate Pooled Probit		Univariate RE Panel Probit	
	Coeff.	Robust SE	Coeff.	SE
Blood pressure screening one year before (t-1)	0.634	(0.024)***	0.480	(0.028)***
Blood pressure screening two years before (t-2)	0.364	(0.026)***	0.217	(0.028)***
Blood pressure screening three years before (t-3)	0.346	(0.024)***	0.186	(0.028)***
Cholesterol screening one year before (t-1)	0.171	(0.039)***	0.186	(0.038)***
Cholesterol screening two years before (t-2)	0.079	(0.042)*	0.106	(0.041)**
Cholesterol screening three years before (t-3)	−0.026	(0.039)	−0.012	(0.041)
Blood pressure screening in 1993			0.102	(0.033)***
Blood pressure screening in 1994			0.116	(0.035)***
Blood pressure screening in 1995			0.195	(0.035)***
Cholesterol screening in 1993			0.051	(0.057)
Cholesterol screening in 1994			−0.068	(0.058)
Cholesterol screening in 1995			−0.048	(0.058)
Averaged Total equivalised HH income/100			0.001	(0.017)
Averaged Living with partner			−0.033	(0.073)
Averaged Number of children in household			−0.003	(0.036)
Averaged Secondary education (ISCED)			0.030	(0.213)
Averaged Tertiary education (ISCED)			−0.041	(0.243)
Averaged employment status part-time or full-time			0.130	(0.072)*
Averaged GP visit during last 12 months			0.189	(0.086)**
Averaged Health status good			−0.097	(0.080)
Averaged Health status fair			−0.212	(0.096)**
Averaged Health status poor			−0.393	(0.170)**
Averaged Health status very poor			−0.547	(0.298)*
Averaged status smoker			0.242	(0.079)***
Averaged Moved residence within Great Britain			−0.061	(0.204)
Averaged age			−0.015	(0.003)***
Averaged blood pressure problems			0.266	(0.078)***
Averaged Diabetes mellitus			0.068	(0.147)
Total equivalised and deflated HH annual income	0.004	(0.006)	−0.001	(0.009)
Living with partner	0.044	(0.029)	0.084	(0.059)
Number of children in household	−0.059	(0.015)***	−0.089	(0.026)***
Secondary education (ISCED)	0.084	(0.031)***	0.037	(0.209)
Tertiary education (ISCED)	0.130	(0.034)***	0.173	(0.237)
Employed part-time or full-time	−0.030	(0.031)	−0.130	(0.045)***
GP visit during last 12 months	0.930	(0.031)***	0.984	(0.033)***
Health status good	0.060	(0.031)*	0.123	(0.037)***
Health status fair	0.209	(0.035)***	0.338	(0.045)***
Health status poor	0.369	(0.051)***	0.569	(0.063)***
Health status very poor	0.531	(0.104)***	0.824	(0.111)***
Status smoking	−0.150	(0.032)***	−0.331	(0.066)***
Moved residence within Great Britain	0.128	(0.050)***	0.133	(0.051)***
Region Scotland	0.017	(0.040)	0.031	(0.053)
Region Wales	−0.065	(0.056)	−0.075	(0.066)
Ethnic non-white	−0.064	(0.121)	−0.019	(0.132)
Age between 30 and 39	−0.036	(0.052)	0.038	(0.062)
Age between 40 and 49	−0.024	(0.055)	0.159	(0.071)**
Age between 50 and 59	0.019	(0.056)	0.380	(0.088)***
Age between 60 and 69	0.088	(0.061)	0.589	(0.108)***
Age between 70 and 79	0.095	(0.066)	0.767	(0.129)***
Age between 80 and above	0.067	(0.077)	0.841	(0.150)***
Female sex	0.104	(0.025)***	0.101	(0.032)***
Blood pressure problems	0.817	(0.036)***	0.823	(0.044)***
Diabetes mellitus	0.347	(0.073)***	0.380	(0.106)***
Constant	−1.803	(0.079)***	−1.482	(0.165)***
σ_α_			0.362	(0.023) ***

Source: BHPS. Balanced panels consisted for blood pressure screening of 1,626 individuals with 21,138 observations. Robust SEs are displayed in

parentheses, to account for individual repeated observations in the panel. *p < 0.1; **p < 0.05; ***p < 0.01.

**Table 4:** Estimates of the univariate pooled and dynamic RE panel probit cholesterol level check model.

	Univariate Pooled Probit		Univariate RE Panel Probit	
	Coeff.	Robust SE	Coeff.	SE
Blood pressure screening one year before (t-1)	0.082	(0.031)***	0.093	(0.033)***
Blood pressure screening two years before (t-2)	−0.050	(0.033)	−0.035	(0.033)
Blood pressure screening three years before (t-3)	0.060	(0.031)*	0.076	(0.033)**
Cholesterol screening one year before (t-1)	1.059	(0.033)***	0.926	(0.036)***
Cholesterol screening two years before (t-2)	0.473	(0.039)***	0.356	(0.038)***
Cholesterol screening three years before (t-3)	0.370	(0.036)***	0.230	(0.038)***
Blood pressure screening in 1993			−0.041	(0.036)
Blood pressure screening in 1994			−0.034	(0.038)
Blood pressure screening in 1995			−0.014	(0.037)
Cholesterol screening in 1993			0.177	(0.052)***
Cholesterol screening in 1994			0.158	(0.054)***
Cholesterol screening in 1995			0.204	(0.052)***
Averaged Total equivalised HH income/100			−0.004	(0.018)
Averaged Living with partner			0.167	(0.087)*
Averaged Number of children in household			−0.030	(0.045)
Averaged Secondary education (ISCED)			0.413	(0.287)
Averaged Tertiary education (ISCED)			0.885	(0.328)***
Averaged employment status part-time or full-time			0.104	(0.080)
Averaged GP visit during last 12 months			0.230	(0.100)**
Averaged Health status good			0.014	(0.094)
Averaged Health status fair			−0.165	(0.107)
Averaged Health status poor			−0.060	(0.167)
Averaged Health status very poor			−0.337	(0.280)
Averaged status smoker			0.181	(0.090)**
Averaged Moved residence within Great Britain			−0.064	(0.241)
Averaged age			−0.035	(0.004)***
Averaged blood pressure problems			−0.056	(0.074)
Averaged Diabetes mellitus			−0.318	(0.133)**
Total equivalised and deflated HH annual income	0.013	(0.007)*	0.009	(0.010)
Living with partner	0.026	(0.030)	−0.094	(0.074)
Number of children in household	−0.069	(0.022)***	−0.087	(0.036)**
Secondary education (ISCED)	0.051	(0.032)	−0.386	(0.283)
Tertiary education (ISCED)	0.110	(0.035)***	−0.775	(0.323)**
Employed part-time or full-time	−0.029	(0.038)	−0.152	(0.055)***
GP visit during last 12 months	0.630	(0.042)***	0.629	(0.046)***
Health status good	0.034	(0.038)	0.047	(0.049)
Health status fair	0.110	(0.042)***	0.176	(0.056)***
Health status poor	0.191	(0.055)***	0.280	(0.071)***
Health status very poor	0.299	(0.088)***	0.432	(0.106)***
Status smoking	−0.059	(0.036)*	−0.200	(0.079)**
Moved residence within Great Britain	0.040	(0.061)	0.022	(0.066)
Region Scotland	0.057	(0.046)	0.061	(0.055)
Region Wales	0.010	(0.058)	0.002	(0.069)
Ethnic non-white	−0.054	(0.145)	−0.051	(0.139)
Age between 30 and 39	0.305	(0.109)***	0.546	(0.119)***
Age between 40 and 49	0.583	(0.110)***	1.131	(0.125)***
Age between 50 and 59	0.709	(0.110)***	1.584	(0.140)***
Age between 60 and 69	0.850	(0.112)***	2.067	(0.160)***
Age between 70 and 79	0.749	(0.116)***	2.280	(0.180)***
Age 80 and above	0.657	(0.120)***	2.479	(0.203)***
Female sex	−0.135	(0.027)***	−0.179	(0.033)***
Blood pressure problems	0.470	(0.032)***	0.559	(0.041)***
Diabetes mellitus	0.533	(0.059)***	0.834	(0.095)***
Constant	−2.797	(0.125)***	−2.001	(0.204)***
σ_α_			0.300	(0.032)***

Source: BHPS. Balanced panels consisted for cholesterol level check of 1,626 individuals with 21,138 observations. Robust SEs are displayed in parentheses, to account for individual repeated observations in the panel. *p < 0.1; **p < 0.05; ***p < 0.01.

In order to obtain valid estimates for the univariate dynamic RE panel probit estimation in comparison to the bivariate estimation the implicit assumptions was made that the correlation of the individual specific random effects terms across the 2 equations was equal to 0. In addition, it is assumed that the coefficients of the cross-lagged variables for the screening examinations were equal to 0. The estimation results for the uptake of blood pressure check and cholesterol level check with the bivariate pooled probit model and the dynamic random effects bivariate panel probit with initial conditions estimator are given in Table 5. The significant own- and cross-lagged dependent variable effects were evident both in the dynamic bivariate pooled probit model and in the bivariate dynamic random RE panel probit model. In the blood pressure check equation, the coefficient for the first order own-effect lagged dependent variable was 0.480, the second order own-effect lagged dependent variable was 0.218 and the coefficient for the third order own-effect lagged dependent variable was 0.188 for the bivariate Wooldridge-type estimators. Similar results exist in the cholesterol level check equation for the first order own-effect lagged dependent variable with a coefficient of 0.924, a coefficient of 0.357 for the second order own-effect lagged dependent variable and a coefficient of 0.227 for the third order own-effect lagged dependent variable. Also significant results had been for the cross-order lagged dependent variables: the first and second order lagged cholesterol level check dependent variable were 0.182 and 0.111 in the blood pressure check equation. The first and third order lagged blood pressure check coefficient were 0.099 and 0.077 in the cholesterol level check equation. The dynamic bivariate pooled probit model and the dynamic RE bivariate panel probit showed similar coefficients for the socioeconomic variables and the coefficients for the lagged dependent variables were higher in the pooled probit models.

**Table 5:** Estimates of the bivariate pooled and dynamic RE panel probit blood pressure check and cholesterol level check model.

	Bivariate Pooled Probit		Bivariate Pooled Probit		Bivariate RE Panel Probit		Bivariate RE Panel Probit	
	Blood pressure check		Cholesterol level check		Blood pressure check		Cholesterol level check	
	Coeff.	Robust SE	Coeff.	SE	Coeff.	Robust SE	Coeff.	Robust SE
Blood pressure screening one year before (t-1)	0.630	(0.024)***	0.094	(0.031)***	0.480	(0.027)***	0.099	(0.035)***
Blood pressure screening two years before (t-2)	0.360	(0.026)***	−0.050	(0.033)	0.218	(0.028)***	−0.042	(0.034)
Blood pressure screening three years before (t-3)	0.344	(0.024)***	0.071	(0.030)**	0.188	(0.027)***	0.077	(0.034)**
Cholesterol screening one year before (t-1)	0.166	(0.038)***	1.057	(0.033)	0.182	(0.040)***	0.924	(0.036)***
Cholesterol screening two years before (t-2)	0.083	(0.041)**	0.472	(0.039)	0.111	(0.042)***	0.357	(0.038)***
Cholesterol screening three years before (t-3)	−0.023	(0.038)	0.366	(0.035)***	−0.008	(0.042)	0.227	(0.039)***
Blood pressure screening in 1993					0.103	(0.033)***	−0.028	(0.036)
Blood pressure screening in 1994					0.114	(0.035)***	−0.033	(0.038)
Blood pressure screening in 1995					0.190	(0.035)***	−0.014	(0.037)
Cholesterol screening in 1993					0.043	(0.056)	0.165	(0.052)***
Cholesterol screening in 1994					−0.055	(0.057)	0.166	(0.054)***
Cholesterol screening in 1995					−0.064	(0.058)	0.198	(0.052)***
Averaged Total equivalised HH income/100					0.000	(0.017)	−0.001	(0.018)
Averaged Living with partner					−0.017	(0.073)	0.174	(0.087)**
Averaged Number of children in household					−0.005	(0.036)	−0.011	(0.045)
Averaged Secondary education (ISCED)					0.033	(0.213)	0.372	(0.292)
Averaged Tertiary education (ISCED)					−0.032	(0.243)	0.864	(0.331)***
Averaged employment status part-time or full-time					0.121	(0.071)*	0.094	(0.079)
Averaged GP visit during last 12 months					0.185	(0.085)**	0.244	(0.100)**
Averaged Health status good					−0.095	(0.079)	−0.003	(0.093)
Averaged Health status fair					−0.218	(0.095)**	−0.190	(0.106)*
Averaged Health status poor					−0.378	(0.168)**	−0.089	(0.167)
Averaged Health status very poor					−0.545	(0.289)*	−0.348	(0.279)
Averaged status smoker					0.264	(0.078)***	0.190	(0.089)**
Averaged Moved residence within Great Britain					−0.060	(0.201)	0.012	(0.240)
Averaged age					−0.016	(0.003)***	−0.034	(0.004)***
Averaged blood pressure problems					0.233	(0.077)***	−0.048	(0.074)
Averaged Diabetes mellitus					0.110	(0.145)	−0.304	(0.132)**
Total equivalised and deflated HH annual income	0.004	(0.006)	0.011	(0.007)	−0.002	(0.009)	0.006	(0.010)
Living with partner	0.043	(0.029)	0.036	(0.030)	0.074	(0.059)	−0.091	(0.074)
Number of children in household	−0.059	(0.015)***	−0.071	(0.022)***	−0.089	(0.026)***	−0.101	(0.036)***
Secondary education (ISCED)	0.086	(0.031)***	0.048	(0.031)	0.037	(0.209)	−0.349	(0.289)
Tertiary education (ISCED)	0.132	(0.034)***	0.101	(0.035)	0.165	(0.237)	−0.764	(0.325)**
Employed part-time or full-time	−0.030	(0.031)	−0.027	(0.038)	−0.124	(0.045)***	−0.145	(0.055)***
GP visit during last 12 months	0.930	(0.031)***	0.632	(0.041)***	0.983	(0.033)***	0.633	(0.046)***
Health status good	0.061	(0.031)*	0.020	(0.038)	0.122	(0.037)***	0.039	(0.048)
Health status fair	0.210	(0.035)***	0.103	(0.042)**	0.337	(0.045)***	0.175	(0.056)***
Health status poor	0.367	(0.050)***	0.186	(0.055)***	0.564	(0.063)***	0.282	(0.070)***
Health status very poor	0.552	(0.103)***	0.287	(0.087)***	0.847	(0.110)***	0.432	(0.105)***
Status smoking	−0.151	(0.032)***	−0.062	(0.036)*	−0.343	(0.066)***	−0.209	(0.078)***
Moved residence within Great Britain	0.128	(0.049)***	0.035	(0.060)	0.132	(0.051)***	0.012	(0.066)
Region Scotland	0.021	(0.040)	0.051	(0.046)	0.038	(0.053)	0.055	(0.055)
Region Wales	−0.065	(0.056)	0.008	(0.058)	−0.076	(0.065)	−0.002	(0.069)
Ethnic non-white	−0.098	(0.120)	−0.011	(0.142)	−0.056	(0.128)	−0.018	(0.136)
Age between 30 and 39	−0.038	(0.052)	0.279	(0.108)***	0.041	(0.062)	0.529	(0.117)***
Age between 40 and 49	−0.025	(0.055)	0.543	(0.108)***	0.166	(0.071)**	1.092	(0.124)***
Age between 50 and 59	0.018	(0.056)	0.667	(0.108)***	0.390	(0.088)***	1.540	(0.139)***
Age between 60 and 69	0.091	(0.061)	0.817	(0.110)***	0.607	(0.108)***	2.026	(0.159)***
Age between 70 and 79	0.084	(0.065)	0.718	(0.114)***	0.773	(0.129)***	2.235	(0.179)***
Age 80 and above	0.062	(0.077)	0.629	(0.118)***	0.860	(0.150)***	2.431	(0.201)***
Female sex	0.104	(0.025)***	−0.108	(0.026)***	0.104	(0.031)***	−0.150	(0.033)***
Blood pressure problems	0.803	(0.036)***	0.464	(0.031)***	0.820	(0.044)***	0.554	(0.041)***
Diabetes mellitus	0.333	(0.070)***	0.536	(0.059)***	0.348	(0.105)***	0.829	(0.095)***
Constant	−1.798	(0.079)***	−2.779	(0.123)***	−1.468	(0.163)***	−2.040	(0.203)
σ_α1_					0.356		(0.022) ***	
σ_α2_					0.305		(0.032) ***	
ρ	0.584		(0.024)***		0.617		(0.025) ***	
ρ_α_					0.274		(0.107) ***	

Source: BHPS. Balanced panels consisted for blood and cholesterol screening of 1,626 individuals with 21,138 observations. Robust SEs are displayed in parentheses, to account for individual repeated observations in the panel. *p < 0.1; **p < 0.05; ***p < 0.01.

The results of the bivariate Wooldridge-type estimator show that the two equations cannot be estimated separately, because the coefficients for the first-order lagged dependent variables of the other type of screening examination were not 0 and the error terms of both equations were correlated with a value of 0.617 as given by *ρ*. Also the individual specific random effects terms were correlated, because *ρ*_*a*_ had a value of 0.274. Higher uptake for blood pressure check and cholesterol level check examinations was observed for individuals who visited their GP within the previous year, poorer self-perceived health status and existing hypertension and diabetes mellitus. A lower uptake was observed for smoking and employed individuals and individuals with a higher number of children in the household. Also, the uptake for both screening examinations increased with a higher age. Females had a higher uptake for the blood pressure check and a lower uptake for the cholesterol level check. Both screening examinations were not influenced by education level, ethnicity and the level of household income or living with a partner in the same way.

## Discussion

This analysis of the BHPS investigated for the first time the determinants of the screening uptake for blood pressure check and cholesterol level check and possible spillover effects. A dynamic random effects bivariate panel probit model was used for the estimation over a period for 13 years from 1996 to 2008. This statistical model uses lagged dependent variables as predictors and further control variables The uptake of blood pressure check and cholesterol level check was modelled with lagged dependent variables up to order 3 and it was controlled for individual heterogeneity. Economic and non-economic variables have been used as determinants for explaining the screening behaviour, however factors such as motivation and effort exerted for screening could not be measured for an individual and are part of the individual heterogeneity.

Past screening examinations of the same type have a high significant effect of own order lags for blood pressure check and cholesterol level check. These results can be interpreted as adherence to the screening recommendations for these cardiovascular screening examinations and also as persistence in screening behaviour and state dependence^49^. Initial conditions are relevant for both types of health check-ups. Additionally, the empirical analysis showed the existence of cross-lagged dependent variable effects for one type of screening examinations on the other type of examination, i.e. the spillover effect of a cholesterol level check on blood pressure check and vice versa. A spillover effect has also been found for the breast and cervical cancer screening examination in Great Britain^6^. This spillover effect could be explained by the fact that an individual is more accessible for preventive information if one of the cardiovascular screening examination has been done in the past or the individual is advised also to do the other type of cardiovascular screening examination. A further possibility exists that unobserved variables with a potential causal relationship such as motivation and cardiovascular risk factors such as serum glucose levels and tricglyceride levels could be correlated with lagged dependent variables of both types of screening examinations, but this information has been not available in our dataset. Unobserved heterogeneity among individuals can only be controlled in a longitudinal study such as ours. In this study unobserved individual heterogeneity played a role both for blood pressure check and cholesterol level check and it is responsible for about one third of the unsystematic variation in each of the equations. The two individual specific terms of the screening uptake equations are correlated and as a consequence persistent unobserved characteristics such as motivation and effort of individuals not directly observed by the researcher can influence the uptake of the cardiovascular screening examination in both screening uptake equations at the same time. The error terms of both equations are correlated and idiosyncratic events and shocks in a certain period can influence the uptake of both cardiovascular screening examinations. The significant correlation of individual specific random terms, the significance of the coefficients for the cross-lagged dependent variables and the correlation of the error terms show that both screening uptake equations are simultaneously determined. As a consequence, both screening processes are interrelated^50^. Therefore, the blood pressure check and the cholesterol level check cannot be analysed as separate independent equations and both equations have to be estimated jointly. The uptake of both types of cardiovascular screening examination will be influenced if the screening recommendation for one type of cardiovascular examination is changed.

On one hand, blood pressure check and cholesterol level check are influenced by the same health related variables. The uptake of both cardiovascular screening examination increases for example with a worsening self-rated health status. Health status can be interpreted as a proxy for the health stock of an individual^51^ and persons with a poor self-assessed health status will have a higher demand for these two cardiovascular screening examinations in comparison to individuals with a better health status. The increased uptake of the blood pressure check and cholesterol level check is justifiable for individuals with existing diseases such as hypertension and diabetes mellitus, because these screening examinations allow better control of the related cardiovascular risk factors high blood pressure and hypercholesterolemia and they are also recommended by the relevant NICE guidelines^1,2^. Smoking individuals, who are generally perceived to have a tendency for a risky behaviour, show a decreased uptake of both screening examinations. Consistent with this finding, another study also showed a reduced uptake of screening examinations by smokers^52^.

On the other hand, individual and household characteristics such as education, living with a partner, had a different effect or no effect on the uptake of both screening examinations and only the number of children in the household and employment had a negative effect on both types of screening examinations. This result is in agreement with two reviews. The first systematic review analysed the role of socioeconomic determinants for the uptake of different cancer screening examinations and none of the analysed socioeconomic variables had the same influence in all cancer screening examinations^17^. The second review which analysed why individuals did not attend general health checks showed also that no socioeconomic variable influenced all analysed health-check-ups in the same way^18^.

Several limitations restrict the analysis. First, there was no information about results from previous blood pressure checks and cholesterol level checks available. Second, taking part in blood pressure checks and cholesterol level check examinations is self-reported and results could be influenced by a recall bias^53^. Third, data on personal or family history of hypertension or familial hypercholesterolemia was not available in the BHPS and cultural UK medical practices have been not recorded as part of the BHPS. For instance, individuals with a family history of cardiovascular diseases belong to a high risk population with an increased probability of a cardiovascular screening examination. Fourth, there was no information about individuals’ trust in the GP or about the GP practice available. Fifth, no detailed microgeographic information was available and uptake rates for screening examinations can be higher in less deprived areas^54^. Sixth, mean reversion effects could also play a role and could be investigated in future research^55^.

Our findings have important practical implications about health check-ups in the UK with regard to the two cardiovascular screening examinations. The results suggest that a certain group of individuals need to be targeted to boost screening uptake. These include smokers, families with children and employees who showed a significantly lower propensity to have both checks. For all, currently neglected screening for these key cardiovascular conditions will have an important and often adverse long-term health consequences. In turn, this will have aggregate and macroeconomic level ramifications of productivity which will be lower in the presence of morbidity that could be avoided if preventive measures are taken in time.

## Data and Methods

The BHPS which is an annual survey of households in the UK is used for the analysis of blood pressure check and cholesterol level check. This survey involves a national representative sample of more than 5,000 households and included individuals have to been at least 16 years old^56^. The survey began in 1991 and all the original individuals were interviewed annually unless they dropped out of the survey. In the analysis and construction of the balanced sample, only individuals from England, Scotland and Wales were selected, because data collection had not started in Northern Ireland until wave 11. For the construction of the balanced panel, 13 years of information were used: from 1996 to 2008, because the minority of individuals were interviewed in 1991 and the minority of individuals were interviewed for the first time in 1992.

Blood pressure check and cholesterol level check information was available over the entire panel period. Questions about participating in blood pressure check and cholesterol level check were in every wave from the start of the panel survey until 2008. For individuals to be included in the analysis, provision had to be from the NHS; individuals with private provision or with NHS and private provision for these health check-up have been excluded from the analysis. The dependent variable takes the value of 1 in a specific year if the blood pressure check or cholesterol level check was done and 0 otherwise.

In the analysis, age groups were categorized for both screening examination in the following way: 16 to 39 (reference category), 40 to 49, 50 to 59, 60 to 69, 70 to 79, age 80 and over. Household income was deflated and expressed in per capita income terms using the modified OECD scale to adjust for differences in household composition^57^. Actual income was defined as the total equivalised and deflated household annual income divided by 100 in the actual wave and averaged (permanent) household income was defined as annual household income over the panel period. The International Standard Classification of Education (ISCED) was used for the categorisation of educational levels into tertiary, secondary and primary education (reference category). Health status was self-rated and included in the analysis with categories from excellent (1) as reference category, good (2), fair (3), poor (4) to very poor (5)^58^.

A dynamic random effects (RE) panel probit model is used to estimate the uptake of both screening examinations over the panel period from 1996 to 2008. It is possible to model and estimate the effect of state dependence from the same type of screening examination and spillover effects from the other type of screening examination. At the same time such a model also allows to control for unobserved individual heterogeneity (e.g. motivation and effort exerted for screening) and to model the correlation between the individual specific random effects terms. Dynamic spillover effects can exist in a bivariate model from one type of screening examination (e.g. blood pressure check) to the other type of screening examination (e.g. cholesterol level check) and equally the direction of impact which we define here as a spillover effect can go from cholesterol level test to blood pressure check. The influence of household and individual characteristics on the uptake can also be analysed by a dynamic random effects panel probit model.

The dynamic random effects univariate panel probit model can be extended to the dynamic random effects bivariate panel probit model (Alessie *et al*.^59^ and Devicienti *et al*.^50^) and this model has been used to analyse the simultaneous uptake of the breast and cervical cancer screening examination^6^ and also in further panel data applications^60–62^. The Wooldridge estimator specifies a relationship between the unobserved time-invariant individual effect and the observed characteristics and has the assumption of initial conditions. The univariate case for the Wooldridge estimator is defined by the following 3 equations^63^.

1 $${y}_{it}^{\ast }=y{^{\prime} }_{i,t-1}\gamma +x{^{\prime} }_{it}\beta +{c}_{i}+{\mu }_{it}$$

2 $${c}_{i}={a}_{0}+{a}_{1}{y}_{i1}+{\bar{X}}_{i}^{\text{'}}{a}_{2}+{\alpha }_{i}$$

3 $${y}_{it}=\{\begin{array}{c}1,if\,{y}_{it}^{\ast } > 0\\ 0,\,otherwise\end{array}\,\,t=2,\mathrm{...},\,T$$

The first equation defines ${y}_{it}^{\ast }$ as an unobserved latent variable of an individual *i* at a given time *t* who will take part in a specific screening examination, *y*_*i,t-1*_ represents the screening examination decision of an individual *i* in period *t-1*, *γ* is the coefficient for this specific variable. The variable *x* represents a vector of time variant (e.g. income) and time invariant (e.g. ethnicity) covariates, *β* represents the vector of the estimated coefficients which are associated with these covariates. The random error term ${u}_{it}$ of an individual *i* in period *t* is given by normal distribution with zero mean and unit variance. The second equation, ${c}_{i}$ represents the individual specific random effect which is modelled according to equation (2) and ${\bar{X}}_{i}$ represents longitudinal averages of an individual *i* for specified variables. Parameters *α*_0_, *α*_1_, *α*_2_ have to be estimated and *α*_0_ is a term with normal distribution with zero mean and variance ${\sigma }_{\alpha }^{2}$. Typically, a normal density for the individual specific random effect is assumed. Time-varying variables of an individual and the individual specific random effect can be correlated and this possibility is modelled in equation (2) by including the average of these variables over the panel observation period^64^. The third equation represents the observed binary outcome *y*_*it*_ of taking part in one specific screening examination for an individual *i* in period *t*.

The estimation of such a dynamic random effects panel probit model for one dependent variable can be extended to the bivariate case in the following way:

4 $${y}_{1it}^{\ast }={\gamma }_{11}{y}_{1i,t-1}+{\gamma }_{12}{y}_{2i,t-1}+x{^{\prime} }_{it}{\beta }_{1}+{c}_{1i}+{\mu }_{1it}$$

5 $${y}_{2it}^{\ast }={\gamma }_{21}{y}_{2i,t-1}+{\gamma }_{22}{y}_{2i,t-1}+x{^{\prime} }_{it}{\beta }_{2}+{c}_{2i}+{\mu }_{2it}$$

6 $${\rm{with}}\,{y}_{jit}=1+[{y}_{jit}^{\ast } > 0]j=1,2\,t=2,\mathrm{...},T$$

${y}_{jit}^{\ast }$ represents the chance of an individual *i* in period *t* to have a screening examination *j* and is expressed as a latent variable. The vector $\beta =({\beta }_{1},\,{\beta }_{2})$ represents the estimated coefficients which are associated with the covariates in equations (4) and (5). It is typically assumed that the error terms ${\mu }_{1it}$ and ${\mu }_{2it}$ have a bivariate normal distribution with a zero mean and a unit variance for each of the error terms and also independence over time and a cross-equation covariance of *ρ* is assumed. The individual specific random effect terms are given by ${c}_{1i}$ and ${c}_{2i}$ for the blood pressure check and the cholesterol level check and a bivariate normal distribution is assumed. Variances of these terms are given by ${\sigma }_{c1}^{2}$ ${\sigma }_{c2}^{2}$ and covariances are given by ${\sigma }_{c1}$, ${\sigma }_{c1}$, ${\rho }_{c}$ in such a model.

The equation (6) represents the observed binary outcome of an individual *i* in period *t*, for the blood pressure check (${y}_{1it}$) and is equal to 1 if the individual *i* has a blood pressure check in period t and 0 otherwise. In the same way, the observed binary outcome for the individual *i* in period *t* for the cholesterol level check (${y}_{2it}$) is equal to 1 if an individual *i* has a cholesterol level check in period t and 0 otherwise.

The inclusion of lagged dependent variables for blood pressure check and cholesterol level check gives the possibility to make a distinction between unobserved individual heterogeneity and state dependence possible. It allows also to analyse potential dynamic spillover effects from one type of screening examination to the other type of screening examination. The chosen specification allows for correlated unobserved individual heterogeneity between the two processes and takes into account the initial conditions of each screening examination. It is also possible to analyse if the correlation of the observed outcomes for blood pressure check and cholesterol level check is caused by correlation of unobserved individual heterogeneity (${\rho }_{c}\ne 0$) or by spillover effects between both screening examinations (${\gamma }_{12}$ and ${\gamma }_{21}$ $\ne$ 0). The dynamic random effects bivariate panel probit model which is given below by equations (7) and (8) can be simplified in certain cases. If the coefficients ${\gamma }_{12}$ and ${\gamma }_{21}$ are both 0, both equations could be estimated independently and so each of the equations could be estimated as a dynamic random effects univariate panel probit model. If the coefficients ${\gamma }_{12}\ne 0$ or ${\gamma }_{21}\ne 0$ and the individual specific random effects and the error terms of both equations are independent, which requires $\rho =0$ and, ${\rho }_{c}=0$, then equation (7) or equation (8) could also be estimated as separate equations. Both equations have to be estimated jointly for getting consistent estimates if these conditions are not fulfilled.

7 $${y}_{1it}^{\ast }={\gamma }_{11}{y}_{1i,t-k}+{\gamma }_{12}{y}_{2i,t-k}+x{^{\prime} }_{it}{\beta }_{1}+{a}_{10}+{a}_{11}{y}_{1i1}^{^{\prime} }+{a}_{12}{y}_{2i1}^{^{\prime} }+{\bar{X}}_{i}^{^{\prime} }{a}_{13}+{\alpha }_{1i}+{u}_{1it}$$

8 $${y}_{2it}^{\ast }={\gamma }_{21}{y}_{1i,t-k}+{\gamma }_{22}{y}_{2i,t-k}+x{^{\prime} }_{it}{\beta }_{2}+{a}_{20}+{a}_{21}{y}_{1i1}^{^{\prime} }+{a}_{22}{y}_{2i1}^{^{\prime} }+{\bar{X}}_{i}^{^{\prime} }{a}_{23}+{\alpha }_{2i}+{u}_{1it}$$

with

9 $${c}_{1i}={a}_{10}+{a}_{11}{y}_{1i1}^{^{\prime} }+{a}_{12}{y}_{2i1}^{^{\prime} }+{\bar{X}}_{i}^{^{\prime} }{a}_{13}+{\alpha }_{1i}$$

10 $${c}_{2i}={a}_{20}+{a}_{21}{y}_{1i1}^{^{\prime} }+{a}_{22}{y}_{2i1}^{^{\prime} }+{\bar{X}}_{i}^{^{\prime} }{a}_{23}+{\alpha }_{2i}$$

The Wooldridge estimator for the bivariate case is estimated using a simulated Maximum Likelihood estimator with 2RN Halton draws. N is the number of individuals and R defines the number of replications; 60 replications were used for the final estimation. The Alessie estimator^59^ which extends the Heckman estimator^65^ to the bivariate case for the dynamic random effects panel probit is an alternative estimation method. However, the Wooldridge estimator has certain advantages in comparison to the Alessie estimator: the numbers of estimated parameters are smaller and higher order dynamics with lagged dependent variables can be more easily modelled and because of these reasons the Wooldridge estimator was the preferred one for the estimation. In addition, simulation studies show that none of the estimators dominate the other in all respects even if each has its own merits^66^. More importantly, it was found that the two models provide similar coefficient estimates and hence changing an estimator will not lead to significant qualitative changes to the estimates. In this study, the chosen dynamic specification the Wooldridge estimator with 1-year, 2-year, 3-year lagged dependent variables as explanatory variables takes into account the existence of recommendations for blood pressure check and cholesterol level check. Also, screening examinations from the previous year could have a borderline result with the consequent need for further cardiovascular screening examinations. The information about taking part in both cardiovascular health check-ups from 1993 to 1995 have been used for the initial conditions of both screening examinations.

Relevant assumptions for the estimation of the dynamic random effects univariate and bivariate panel probit model are the following ones: the distributional assumptions on the initial conditions are specified in a correct way and the relationship between the unobserved time-invariant individual effect and the mean of the observed characteristics has been correctly specified. A further assumption for an unbiased estimation with regard to the initial conditions is the requirement that unobserved blood pressure check and cholesterol level check examinations that happened prior to the panel observation period are uncorrelated with the observed blood pressure check and cholesterol level check examinations. If these assumptions are violated the estimation results could be biased. It is assumed for the estimation that screening examinations which had been undertaken before the first wave of the BHPS are uncorrelated with the screening examinations recorded in the BHPS. If this assumption is violated, the inclusion of initial conditions of health check-ups for the years 1993 to 1995 could result in biased estimates for the regressions.

The dynamic random effects univariate and bivariate panel probit models are estimated for blood pressure check and cholesterol level check with lagged dependent variables as explaining variables and lags were used up to order 3. In a first step, a dynamic univariate pooled probit with the assumption of exogenous lags was applied and the dynamic random effects univariate panel probit for the blood pressure check and cholesterol level check were estimated. In a second step, a dynamic bivariate pooled probit model and a dynamic random effects bivariate panel probit were estimated for both types of screening examinations. These estimations of the bivariate probit models were compared with the estimations for the univariate probit models and also both bivariate probit models were compared with each other. It is necessary to rescale the coefficients for comparing the coefficients of the dynamic random effects panel probit and the pooled panel probit model, because the coefficients have different normalizations: the coefficients of the dynamic random effects model have to be multiplied by ${(1-{\sigma }_{\alpha }^{2})}^{-1/2}$ if they are compared with dynamic pooled probit model.

### Data availability

The dataset is from a third party (UK Data Archive). The restrictions prohibit the authors from making the minimal data set publicly available. Data can be requested from the UK Data Archive and syntax of the statistical software programmes can also be requested from the UK Data Archive.
